# Pharmacogenetic variation of *SLC47A1* gene and metformin response in type2 diabetes patients

**Published:** 2017-06

**Authors:** Saeedeh Mousavi, Leila Kohan, Majid Yavarian, Asadollah Habib

**Affiliations:** 1 Department of Biology, Arsanjan branch, Islamic Azad University, Arsanjan, Iran; 2 Yong researchers and elite club, Islamic Azad University, Arsanjan Branch, Arsanjan, Iran; 3 Shiraz Nephro-Urology Research Center, School of Medicine, Shiraz University of Medical Sciences, Shiraz, Iran; 4 Department of Endocrinology, School of Medicine, Shiraz University of Medical Sciences, Shiraz, Iran

**Keywords:** Diabetes, Pharmacogenetics, *SLC47A1*, Polymorphism

## Abstract

Type 2 diabetes mellitus is a worldwide epidemic disorder with considerable health and economic consequences. Metformin is one of the most commonly prescribed oral antidiabetic drugs. Pharmacogenetic studies showed that variants in genes related to the pharmacokinetics of metformin were associated with glucose-lowering effect of metformin. The aim of this study was to evaluate pharmacogenetic variation in *SLC47A1* (rs2289669) and metformin response in type 2 diabetes patients. Seventy one patients with type 2 diabetes were included in the study. The genotypes were determined by Tetra–ARMS–PCR method. There was a significant association between the study polymorphism and the response to metformin treatment with the highest HbA1C reduction in AG genotype. In the dominant model for A allele (AA+AG *vs* GG), patients with A allele had highest HbA1C reduction in response to metformin.

## INTRODUCTION

Type 2 diabetes mellitus (T2DM) is a complex metabolic disorder characterized by impaired insulin secretion and insulin resistance [1]. Current therapies for T2DM include lifestyle modification and use of oral antidiabetic drugs [2]. Metformin is one of the most commonly prescribed oral antidiabetic drugs [3]. A recent meta-analysis study showed that metformin monotherapy reduced HbA1c in average by 1.12%. HbA1c is glycated haemoglobin and its level is an indicator of average plasma glucose concentration [4]. A considerable inter-individual variability exists in glucose-lowering response to metformin. Up to one third of patients do not respond adequately to metformin. This interindividual variability can be caused by nongenetic factors such as kidney or liver function and genetic factors such as variation in metformin transporter genes [5]. The *SLC47A1* encodes a member of multidrug and toxin extrusion (MATE) transporter protein family. Metformin is one of the substrates of this transporter [6]. Recently, a noncoding SNP (rs2289669 G/A SNP) of *SLC47A1* has been reported to influence the pharmacodynamic response to metformin, suggesting reduced transport activity of the transporter being associated with a higher reduction in HbA1c [7]. In this study, we studied the association between the *SLC47A1* rs2289669 and metformin response in Iranian patients with T2DM.

## MATERIALS AND METHODS

The 71 diabetes patients who gave informed consent for genetic investigation were the subjects of this study. Patient samples were collected from Bustan clinic in Shiraz. Type 2 diabetes was diagnosed in patients according to the criteria of the American Diabetes Association [8]. Patients with malignancies, chronic kidney disease, endocrine disorders, liver cirrhosis and systemic inflammatory disease were excluded. Drug-naïve patients with HbA1c in the range of 6.5-11% were included. Baseline HbA1c measurement was done within 1 week prior to the treatment initiation and a second measurement after 6 months of metformin monotherapy [9]. The primary study outcomes were based on reduction of HbA1c after 6 months of metformin therapy according to the results of A Diabetes Outcomes Progression Trial (ADOPT) which showed that maximal response to metformin was recorded after the 6-month treatment [10]. *SLC47A1* rs2289669 variant was genotyped by Tetra–ARMS–PCR method [11]. 

Statistical analysis were performed using SPSS 21 software. Continuous variables are presented as mean±standard deviation (SD). For the comparison of continuous variables, unpaired t-test were used. HbA1c response to metformin was compared across different genotypes by one-way ANOVA followed by Fisher's LSD post hoc test. General linear models were used for adjusting values and study of the response of HbA1c to metformin therapy according to genotypes. 

## RESULTS AND DISCUSSION

Seventy one T2DM patients with an average age of 55.1±11.4 years and a mean BMI of 28.1±3.9 kg/m2 were included in the study. Table 1 shows mean level of HbA1c before and after initiation of metformin treatment. The mean baseline level of HbA1c before initiation of metformin treatment was 9.08±1.51%. During first 6 months after initiation of metformin treatment HbA1c level was reduced in the entire study group and the mean decrease of HbA1c (ΔHbA1c) during metformin as a monotherapy was 1.74±1.50%.

**Table 1:** HbA1c level before and after metformin therapy

**Variant**	**Range**	**Mean ** **±** ** SD**
HbA1C	6.1-12.9	9.08±1.51
HbA1C After 6 M	5.3-11.3	7.34±1.22
Δ HbA1C	0.6-6.0	1.74±1.50

The association of the study genotypes and ΔHbA1c is shown in Table 2. The study sample was in Hardy-Weinberg equilibrium (χ^2^=0.50, df=1, P=0.48). We observed significant association between the rs2289669 polymorphism and HbA1c reduction after initiation of metformin therapy. There was a significant difference between SLC47A1 rs2289669 genotype groups in response to metformin. Also, the highest HbA1c reduction was observed in AG genotype.

**Table 2:** Therapeutic response to metformin according to SLC47A1 rs2289669 genotypes

**Polymorphism**	**GG**	**AG**	**AA**	**P (Between groups)**
Patients numbers (%)	24 (33.8)	37 (52.1)	10 (14.1)	
ΔHbA1c (%)	0.91±0.84	2.44±1.51	1.15±1.49	<0.001
P ^LSD^	-	<0.001	0.63	
*P ^Adjusted^				<0.001

* P ^Adjusted^: values after adjustment for age, sex, BMI, baseline HbA1c level and metformin dose in general linear models.

This population-based cohort study in diabetic patients showed that the rs2289669 polymorphism was associated with the HbA1c lowering effect of metformin. Patients with AG genotype had highest HbA1C reduction in response to metformin. Several studies reported a link between *SLC47A1* rs2289669 polymorphism with HbA1c reduction by metformin [7,9,12]. Tkác et al 2012 reported that the homozygous carriers of *SLC47A1* rs2289669 A allele have twofold reduction in HbA1c during the first 6 months of metformin treatment in comparison with the rest of diabetes patients [9]. Also, the current study confirms the results of a preliminary study of Becker et al. who identified 116 metformin users in the prospective epidemiological Rotterdam study and found an increase of metformin effect of 0.3% per one A-allele of this variant [7]. In other study of Chinese patients, The AA homozygous carriers of the *SLC47A1* rs2289669 G>A polymorphism had a lower clearance (P<0.01) of metformin than carriers of the wild type [12]. The mechanism whereby this polymorphism affects metformin action is not elucidated so far, because it is a non-coding intronic variant. It is possible that the A-variant is in linkage disequilibrium with a reduced function variant [9]. To conclude, we found an association between the SNP rs2289669 in the *SLC47A1*, encoding the MATE1 transporter, and the glucose-lowering effect of metformin. These results suggest that MATE1 may have an important role in the pharmacokinetics and pharmacodynamics of metformin. This is the first epidemiological study assessing the role of *SLC47A1* polymorphism in metformin response in Iranian population.
